# A Male Case of Kagami-Ogata Syndrome Caused by Paternal Unipaternal Disomy 14 as a Result of a Robertsonian Translocation

**DOI:** 10.3389/fped.2020.00088

**Published:** 2020-03-10

**Authors:** Xiaoxue Wang, Hui Pang, Birju A. Shah, Hongcang Gu, Lijun Zhang, Hua Wang

**Affiliations:** ^1^Department of Hematology, The First Hospital of China Medical University, Shenyang, China; ^2^Department of Pediatrics, University of Oklahoma Health Sciences Center, Oklahoma City, OK, United States; ^3^Epigenomics Program, Broad Institute of MIT and Harvard, Cambridge, MA, United States; ^4^Division of Genetics, Department of Pediatrics, Loma Linda University School of Medicine, Loma Linda, CA, United States

**Keywords:** uniparental disomy, Kagami–Ogata syndrome, imprinting disorders, Robertsonian translocation, heterodisomy

## Abstract

Kagami–Ogata syndrome (KOS) is a rare imprinting disorder characterized by skeletal abnormalities, dysmorphic facial features, growth retardation and developmental delay. The genetic etiology of KOS includes paternal uniparental disomy 14 [upd(14)pat], epimutations and microdeletions affecting the maternally derived imprinted region of chromosome 14q32.2. More than seventy KOS cases have been reported thus far; however, only 10, including two familial, are associated with upd(14)pat harboring Robertsonian translocation (ROB). Here, we reported a male infant with clinical manifestations of facial dysmorphism, bell-shaped small thorax, and omphalocele. Karyotype analyses identify a balanced ROB involving the long arms of chromosomes 13 and 14 both in the patient and his father. We further confirm the pattern of upd(14)pat utilizing DNA polymorphic markers. In conclusion, our case report provides a new male KOS case caused by upd(14)pat with paternally inherited Robertsonian translocation, which represents the second male case officially reported. Notably, a KOS case due to upd(14)pat and ROB is rare. An accurate diagnosis requires not only the identification of the characteristic clinical features but also systemic cytogenetic and molecular studies.

## Introduction

Genomic imprinting is an epigenetic phenomenon that restricts the expression of genes in an imprinted region to a single parental allele (1). Genomic or epigenetic changes of imprinted regions have been linked to certain genetic disorders (2). So far, six imprinted regions associated with nine clinically recognized imprinting disorders have been reported in human (3, 4). The common causes of imprinting disorders included genomic and epigenetic alterations such as mutations/copy number changes of imprinted genes, uniparental disomy (UPD) and increases or decreases of DNA methylation levels (5).

Kagami–Ogata syndrome (KOS; MIM 608149) is an imprinting disorder involving genes in chromosome 14q32.2 region and paternal uniparental disomy 14 [upd(14)pat] accounts for approximately two-thirds of KOS patients (6). The main clinical features of KOS include craniofacial dysmorphism, thoracic dysplasia (bell-shaped small thorax with coat-hanger appearance), abdominal wall defect, growth retardation, and developmental delay (7). UPD(14)pat has been reported as a *de novo* event as well as a result of a paternally inherited Robertsonian translocation (ROB) or due to a monosomy rescue which leads to paternal isodisomy (8). ROBs are whole-arm exchanges of acrocentric chromosomes which may cause unproperly segregation of homologous chromosomes in meiosis leading to aneuploidy conceptions. Many cases of UPD have been identified in carriers of ROBs and the risk of UPD in carriers of non-homologous ROB is 0.6–0.8% (9, 10). Only one of the ten reported KOS cases caused by ROB related UPD was from a male patient. Parental karyotype was performed on seven patients, and the results showed that five were *de novo*, and the other two were inherited from the father. Here, we presented another male patient with characteristic KOS features caused by upd(14)pat and balanced ROB inherited from the father.

## Case Report

**Figure 1:**
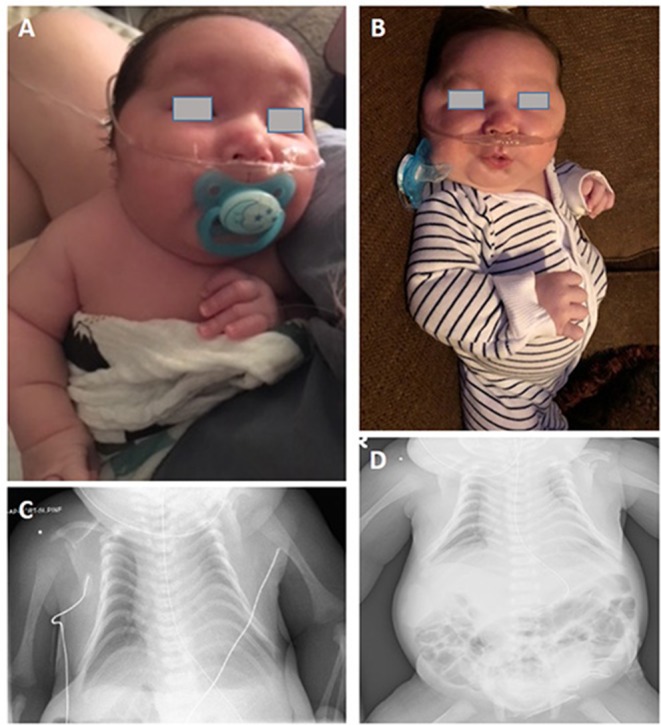
**(A,B)** Photograph of the patient. **(C)** Chest radiography of the patient. Thoracic dysplasia (bell-shaped small thorax with coat-hanger appearance). **(D)** Anterior abdominal wall defect with bowel herniation consistent with history of omphalocele. (The written informed consent was obtained from the parents of the patient for the publication of this image).

**Figure 2:**
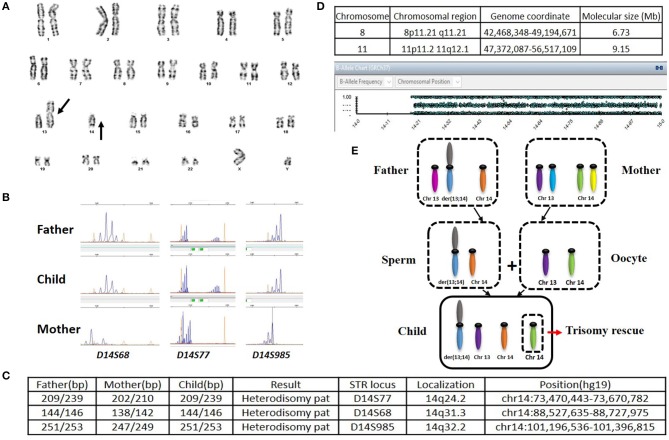
**(A)** Karyotype of the patient. **(B)** Microsatellite analysis. Maternally and paternally derived peaks are showed, respectively. **(C)** The short tandem repeats (STRs) on the long arm of chromosome 14 analyzed along with their position (hg19 map). **(D)** Result of SNP-Array. Homozygous regions in chromosome 8 and 11 were identified (Top); No homozygous region in chromosome 14 was detected (Bottom). **(E)** Schematic representation of the trisomy rescue (TR) mechanism leading to the heterodisomy upd(14)pat. A nondisjunction event between two chromosomes 14 occurred when a male ROB carrier produce a sperm. The segregation of the chromatids determined the formation of sperm with a ROB and a free chromosome 14. Oocyte has one of each normal chromosome 13 and 14. Then, after fertilization, there would be three chromosome 14 in the zygote. In order to avoid cell death of the three chromosome 14, the normal chromosome 14 from the mother will be excluded through the process of post-zygotic TR, which could result in paternal heterodisomy in the early embryo.

This male infant was born at 36 weeks and 6 days of gestation by a cesarean section due to prenatal findings of congenital anomalies and polyhydramnios. The proband was conceived naturally to a 34-year-old father and a 31-year-old mother both of whom are Caucasian origin. The couple had a history of a previous miscarriage. There was no family history of genetic disorders, and parental consanguinity was denied. Ultrasound immediately prior to delivery was significant for omphalocele and amniotic fluid index of 38 (Normal value 5–25). Apgar scores were six and eight at 1 and 5 min of life, respectively. The admission exam was consistent with omphalocele, with a birth weight of 3,870 grams (Z score 2.08, >97th percentile), head circumference of 37 cm (Z score 2.55, >97th percentile), and length of 45 cm (Z score −1.29, 10th percentile). On physical examination at birth, dysmorphic facial features were noted including narrow forehead, frontal bossing, short palpebral fissures, depressed nasal bridge, anteverted nares, elongated philtrum, low-set posteriorly rotated ears, and micrognathia (Figures 1A,B). Additionally, short neck, bell-shaped chest, elongated fifth finger on each hand with flexion contractures, deep sacral dimple, bilateral undescended testes and right hydrocele were identified. There was no cardiac murmur or organomegaly. Echocardiography showed a patent foramen ovale. Abdominal ultrasound showed left-sided mild hydronephrosis, and skeletal survey displayed 11 pairs of ribs and bell-shaped thoracic cavity with coat-hanger looking ribs (Figures 1C,D). Primary repair of omphalocele on day 4 of life was performed under general endotracheal anesthesia. Later, gastrostomy tube was placed due to significant aspiration with penetration of thin liquids on dysphagiogram. Brain and spinal cord MRI showed echogenic scattered foci in periventricular white matter without restricted diffusion. Screening electroencephalogram was unremarkable for seizures. The placental pathology was unremarkable. The infant was discharged home on low-flow nasal cannula with supplemental oxygen, monitor and suction equipment on day 36 of life. Due to his multiple congenital anomalies, routine chromosome analysis was performed on peripheral blood and the results revealed an abnormal male karyotype with a balanced ROB:45,XY,der(13;14)(q10;q10) (Figure 2A). Further parental karyotyping indicated that this ROB chromosome was inherited from his father. The presence of paternally inherited der(13;14) combined with the patient's phenotype raised the concern for KOS caused by upd(14)pat. Therefore, microsatellite analysis was subsequently performed by utilizing multiple polymorphic DNA markers on subchromosomal regions 14q24.2(*D14S77*), 14q31.3(*D14S68*), and 14q32.2(*D14S985*). All three markers demonstrated that the patient inherited two homologous chromosome 14 from his father, confirming a paternal uniparental heterodisomy of 14q (Figures 2B,C). Then, the whole genome SNP array analysis were used to detect if there were additional small deletion or duplication. The test identified two homozygous regions with varied molecular sizes but did not detect any clinically relevant copy number change (Figure 2D). In the constellation of whole clinical picture, the results are consistent with the diagnosis of KOS caused by upd(14)pat. The follow-up investigation showed that the patient was transitioned from utilizing supplemental oxygen to room air. He's following pulmonology for surveillance of respiratory function secondary to thoracic dysplasia, urology for undescended testes and hydronephrosis, and genetics. Furthermore, the GI team is transitioning him to bolus feeding using a gastrostomy tube. He is 20-month-old and seems to be reassuring so far.

## Discussion

Among all the published KOS cases, more than 60% were caused by upd(14)pat, nearly 25% due to by microdeletions of chromosome 14q32 imprinted region, about 10% derived from epimutations (6, 11). The imprinted region on chromosome 14q32 harbor paternally expressed genes such as *DLK1* and *RTL1*, and maternally expressed genes including *MEG3, RTL1as* (*RTL1* antisense) and *MEG8*. Intergenic differentially methylated region (IG-DMR) between *DLK1* and *MEG3* as well as the post fertilization-derived secondary MEG3-DMR are of great importance for controlling imprinting (12, 13). The abnormalities of KOS caused by imprinting defects are due to abnormally expressed dosages of imprinted genes (overexpression of DLK1 and RTL1 as well as underexpression of MEGs). In our and not in this case, the abnormalities are probably originated from the overexpression of genes on the paternally inherited allele. Although phenotypes are comparable among patients with different genetic etiologies, placentomegaly or polyhydramnios may not present among KOS patients with microdeletion (13). Altogether, about 20% of the patients harboring UPD along with chromosome structural anomalies (acrocentric isochromosomes, ROB, marker chromosomes, derivative chromosomes, reciprocal translocations, etc.) displayed non-homologous ROB (14). The potential mechanisms leading to UPD include trisomy rescue (TR), gamete complementation (GC), monosomy rescue (MR), and post-fertilization mitotic error (PE) (15). The underlying mechanism of the strong correlation between ROB and hetero-upd(14)pat in our case is considered to be TR (Figure 2E). Interestingly, the numbers of the particular cases with upd(14)pat harboring ROB are relatively small in the current collection of surveys. Only eleven cases, including this one, have been reported; and most of the patients were female (14, 16–24). To provide a better understanding of KOS cases with upd(14)pat with ROB in particular, we review the published articles and the results are summary in Table 1. Furthermore, among the eight cases with detailed clinical information and karyotypes from both the patient and the parents, three cases present paternally derived ROB (16, 18) and our case is the only male case. The other male case was reported by Walter et al. (22) in 1996. The patient had similar symptoms to our case. It is not clear why the proportion of male patients is low. It is possible that it is due to a bias because of the low numbers. We need to accumulate more cases' information and improve the relevant examinations in order to better understand such disease.

**Table 1:** Published case reports of upd(14)pat with Robertsonian translocation.

**References**	**Location**	**Sex/age**	**Phenotype (common features)**	**Phenotype** **(unique features)**	**Genotype**	**Maternal genotype**	**Paternal genotype**
Wang et al. (16)	USA	Female/9-year-old (at the last examination)	Short neck with webbing, a small thoracic cage causing restrictive lung disease, marked angulation of the ribs, small ears, anteverted nares, protruding philtrum, severe mental retardation, and coarse facial features with frontal bossing and prominent maxilla and mandible	Bilateral subarachnoid hygromas requiring a shunt, bilateral Simian creases, and blepharophimosis, severe kyphoscoliosis, seizure disorder	45,XX,t(13q;l4q)	46,XX,t(1;14) (q32;q32)	45,XX,t(13q;14q)
Papenhausen et al. (17)	USA	Female/20- Second -old (at the last examination)	Puckered lips, hairy forehead, distended abdomen with ventral wall hernia, hypotonia, mechanical ventilation	NA	45,XX,t(14q;14q)	Normal	Normal
Cotter et al. (18)	USA	Female/died 6 months	Polyhydramnios, non-pitting edema, a short neck, and a small, a bell-shaped, short thorax with thin ribs, a depressed nasal bridge, small ears, a protruding philtrum, short palpebral fissures, mechanical ventilation	Ossification defect of the cranial base, short long bones	45,XX,der(13;14) (q10;q10)	45,XX,der(14;21)(q10;q10)	45,XY,der(13;14)(q10;q10)
Kurosawa et al. (19)	Japan	Female/11-month-old (at the last examination)	Polyhydramnios, frontal bossing, hairy forehead, depressed nasal bridge, anteverted nares, protruding philtrum, micrognathia, Small bell-shaped thorax in infancy, coat-hanger, mechanical ventilation, diastasis recti	Blepharophimosis, hepatoblastoma	45,XX,der (13q;14q)	NR	NR
McGowan et al. (20)	USA	Female/died 6 weeks	Polyhydramnios, short neck, mild pectus excavatum, slight peripheral edema, and mechanical ventilation, feeding difficulty	Abnormal flexion of the left thumb	45,XX,der(14;14)(q10;q10)	NR	NR
Kagami et al. (21)	Japan	Female/20-month-old(at the last examination)	Polyhydramnios, placentomegaly frontal bossing, hairy forehead, small ears, depressed nasal bridge, anteverted nares, full cheeks, protruding philtrum, short webbed neck, small bell-shaped thorax in infancy, coat-hanger appearance in infancy, diastasis recti, constipation, development delay, feeding difficulty	Blepharophimosis, laryngomalacia, joint contractures	45,XX,rob(14;21)(q10;q10)	NR	NR
Walter et al. (22)	USA	Male/6-month-old (at the last examination)	Polyhydramnios, digit contractures, small thorax, abnormal ribs, low birth weight, short birth length, required gastrostomy, Small ears, Simian creases Blepharophimosis/short palpebral fissures, protruding philtrum, puckered lips, short limbs	Heart murmur, undescended testes, required tracheostomyl/endotracheal intubation	45,XY,dic(14)(pll)	Normal	Normal
Stevenson et al. (23)	USA	Female/7-month-old (at the last examination)	Short palpebral fissures, epicanthal folds, flat nasal root, anteverted nares, long protruding philtrum, small mouth, retrognathia, redundant nuchal skin folds, prominent diastasis recti, and hypotonia a small and bell shaped thorax, and underdevelopment of the scapular necks, Polyhydramnios, feeding difficulty	plagiocephaly, inverted nipples	45,XX,inv(9)(p11q13),dic(14;14)(p11.1;p11.1)	Normal	46,XY,inv(9)(p11q13).
Berend et al. (14)	USA	Female	Polyhydramnios, contractures	NR	45,XX der(14;14)	Normal	Normal
Berend et al. (24)	USA	Female	Developmental delay, mental retardation, birth defects	NR	45,XX, i(14)(q10)	Normal	Normal
This study	USA	Male/11-month-old(at the last examination)	Polyhydramnios, narrow forehead, frontal bossing, short palpebral fissures, depressed nasal bridge, anteverted nares, elongated philtrum and micrognathia, bell shaped narrow thoracic cavity with coat-hanger looking ribs, omphalocele	Bilateral undescended testes, left mild hydronephrosis, sacral dimple, adducted thumbs, brachydactyly.	45,XY,der(13;14)(q10;q10)	Normal	45,XY,der(13;14)(q10;q10)

*NA, not applicable; NR, not reported*.

## Conclusion

This study provides a new male case of KOS caused by upd(14)pat with paternally inherited ROB. For neonates with characteristic clinical features and ROB, further parental study and molecular studies for upd(14)pat should be considered. KOS due to upd(14)pat and ROB is rare, and cytogenetic and molecular studies can help for the diagnosis.

## Data Availability Statement

All datasets generated for this study are included in the article/supplementary material.

## Ethics Statement

The studies involving human participants were reviewed and approved by Oklahoma University Health Science Center. The patients/participants provided their written informed consent to participate in this study. Written informed consent was obtained from the individual(s), and minor(s)' legal guardian/next of kin, for the publication of any potentially identifiable images or data included in this article.

## Consent

Written informed consent was obtained from the patient's parents for publication of this case report and the accompanying images.

## Author Contributions

XW: wrote the manuscript. HP and HG: responsible for the data analysis. BS: cared for the patient and provided clinical history. LZ: responsible for the revisions. HW: provided genetics consultation service and follow up of the patient, clinical data collection, and responsible for the revisions.

### Conflict of Interest

The authors declare that the research was conducted in the absence of any commercial or financial relationships that could be construed as a potential conflict of interest.
